# MicroRNAs: A Social Network in Diabetic Retinopathy

**DOI:** 10.3390/biom16081199

**Published:** 2026-08-17

**Authors:** Sheila Ngumbi, Mohamed S. Gad, Kara Ye, Sarah Ye, Christie Taylor, Mostafa Mahrous, Manuela Bartoli

**Affiliations:** 1 Department of Ophthalmology, Medical College of Georgia at Augusta University, Augusta, GA 30912, USA; foscash@gmail.com (S.N.); mgad@augusta.edu (M.S.G.); ctaylor9@augusta.edu (C.T.); mmahrous@augusta.edu (M.M.); 2 Department of Medical Histology and Cell Biology, Faculty of Medicine, Mansoura University, Mansoura 35516, Egypt; 3 Department of Ophthalmology, LSU Health, New Orleans, LA 70112, USA; kye@lsuhsc.edu; 4 Department of Ophthalmology, Wayne State University, Detroit, MI 48201, USA; sarah.ye@wayne.edu

**Keywords:** diabetic retinopathy, MicroRNAs (miRNAs), non-coding RNAs, exosomes, angiogenesis, inflammation, oxidative stress, retinal vascular dysfunction, biomarkers, miRNA-based therapeutics

## Abstract

In recent years, the role of non-coding RNAs in human physiology and pathology has emerged as an essential avenue of investigation. Among others, studies identifying the biological role of microRNAs (miRNAs) have paved the way for future inquiries on the importance and biological significance of non-coding RNAs. In this review, we provide an overview of miRNAs’ biology and their contribution to the pathogenesis of diabetic retinopathy (DR). This complication of diabetes is the leading cause of blindness in adults, affecting more than 4 million people in the US alone and over 103 million people worldwide. Despite the tremendous efforts of the scientific community and the pharmaceutical industry, the development of new, more effective therapeutic and diagnostic tools for DR to date remains an unmet need. More than a decade from the initial work assessing miRNA expression profiles in diabetic patients, we have learned the impact of these signaling molecules in DR and garnered knowledge of their complexity and their potential as new diagnostic and therapeutic targets for this potentially blinding condition.

## 1. Introduction

The complexity of the genetic code has been evident since the first identification of DNA in 1860 by Johann Friedrich Miescher and its subsequent characterization in 1950 by James Watson, Francis Crick, and Rosalind Franklin [1]. However, the more recent discovery and characterization of non-coding RNA (ncRNA) species have added a significant layer of complexity underpinning the biological importance of fine-tuned gene regulation and epigenetics.

Dysregulated non-coding RNA expression has been evidenced in pathological conditions and linked these molecular players to the onset and progression of several human pathologies, including cancer, cardiovascular, and neurodegenerative diseases [2].

MicroRNAs (miRNAs) are a single-stranded subset of ncRNAs classified by their length (21–23 nucleotides) and characterized by their gene-silencing ability [3].

The recognition of Viktor Ambros and Gary Ruvkun with the 2024 Nobel Prize in Physiology or Medicine for the discovery of miRNAs highlights the profound significance of these regulatory molecules in shaping our understanding of human biology, physiology, and disease mechanisms [4,5].

In this review, we present an overview of the recent research progress, delving into the role of miRNAs in diabetic retinopathy (DR), a neurovascular complication of diabetes [6], and their potential as diagnostic and therapeutic tools for this potentially blinding condition.

## 2. Diabetic Retinopathy (DR)

### 2.1. Epidemiology

Diabetic retinopathy (DR) is a late complication of Type 1 and Type 2 diabetes (T1D and T2D, respectively) the leading cause of preventable blindness in working-age adults aged 20–74 years old [7]. The development of other diabetic complications, such as diabetic nephropathy, peripheral neuropathy, and cardiovascular events, can be associated with the development and degree of DR [8,9].

The onset of DR correlates with the duration of diabetes, with approximately all patients with T1DM and over 70% of patients with T2DM developing DR by 20 years into the disease. Recent advances in diabetes management have improved glycemic control and are expected to delay disease onset and progression; nevertheless, DR remains a major cause of vision loss worldwide [10,11,12]. Retinopathy is pervasive among patients with T1DM and T2DM in the United States, with a prevalence of 40% (estimated 4 million people) and 80% (estimated 750 thousand people), respectively [13,14]. A high prevalence of DR has been reported in other countries, such as the United Kingdom, with those of South Asian ethnicity being affected in higher proportions than white Europeans [15]. People of Chinese descent are also disproportionately affected by DR, with rates as high as 43%, and 1.2 million of whom have vision-threatening retinopathy [16]. DR incidence and related vision loss is higher in non-Hispanic Black and Hispanic patients compared to Caucasian patients [17]. The National Eye Institute indicates that over 800,000 African Americans currently have DR, with this number expected to rise by 2030 [18].

Recent epidemiological estimates highlight the increasing global burden of DR in parallel with the rising prevalence of diabetes worldwide [19]. The International Diabetes Federation estimates that more than 500 million adults currently live with diabetes, a number projected to substantially increase over the coming decades, contributing to a continued rise in diabetes-associated complications, including DR [20]. Recent meta-analyses estimate that approximately one-third of individuals with diabetes develop some degree of DR, with a substantial proportion progressing to vision-threatening forms, including diabetic macular edema and proliferative diabetic retinopathy [21]. Despite advances in glycemic control and retinal therapies, DR remains a leading cause of preventable vision impairment among working-age adults and represents an increasing public health challenge worldwide [22,23]. Clearly, DR is a significant public health problem not only in the United States but also around the world in high-income and rural areas.

### 2.2. DR Pathogenesis

The pathogenesis of DR is complex, and the molecular mechanisms underlying this pathology are not completely understood. Chronic hyperglycemia affects both vascular and neural tissue in the retina. Persistent hyperglycemia-induced changes in cellular metabolism contribute to oxidative injury of the retinal microvasculature [24,25]. Early changes include loss of retinal supporting pericytes and basement membrane thickening in retinal capillaries [26]. Oxidative damage to pericytes contributes to endothelial dysfunction and increased capillary permeability, leading to consequent macular edema [27]. Weakened vessel walls are more susceptible to forming microaneurysms that are prone to rupture into a dot-blot hemorrhage, and even microvascular thrombosis leading to retinal vessel occlusion and retinal nerve infarction [28].

As the disease progresses, hypoxia-induced growth factors, such as vascular endothelial growth factor (VEGF), are stimulated by reduced blood flow and chronic retinal ischemia due to capillary occlusion [29,30]. The release of these growth factors stimulates retinal neovascularization and the growth of aberrant, fragile blood vessels that tend to hemorrhage. These misdirected blood vessels can grow into the vitreous and hemorrhage or cause tractional retinal detachment, both of which can lead to sudden vision loss [31]. Inflammatory markers such as IL-1β, IL-18, and caspase-1 are important for sterile inflammation via the NLRP3 inflammasome and are elevated in the vitreous of diabetic rat models. Protein expression levels of adhesion molecules ICAM-1 and VCAM-1 and the inflammatory cytokine HMGB1 are higher in the ganglion cell and retinal nerve fiber layer of diabetic patients compared to normoglycemic controls, indicating increased leukocyte infiltration and inflammation [32].

The goal of DR screening is the timely detection of retinal damage and intervention with laser photocoagulation or intraocular glucocorticoids and anti-vascular endothelial growth factor (VEGF). Current screening methods for diabetic DR include direct visualization of signs of microaneurysms, exudates, and intraretinal hemorrhages through dilated fundus exams or retinal photography [33,34]. Additional tests to detect vision-threatening macular edema include fluorescein angiography and optical coherence tomography. Unfortunately, these methods require the expertise of a trained eye care specialist with specialized equipment, and they are only able to diagnose DR once retinal damage has already occurred. Emerging research suggests that several miRNAs may be involved in the development of DR, and these could represent biomarkers to enable better screening and treatment for diabetic individuals at risk of retinopathy [35,36,37].

## 3. MicroRNAs (MiRNAs)

### 3.1. Biogenesis and Function

MiRNAs are small, noncoding RNAs that play a regulatory role in every cell process, including cell development, signaling, proliferation, and differentiation [38,39]. By binding complementary sequences in the 3′ untranslated region of target mRNA, miRNAs can decrease the translation of expressed transcripts [40]. In addition to the miRNA seed sequence, a prominent distinguishing feature of mRNA targets is that miRNAs have imperfect complementarities to the sequence of their target mRNA [41]. This imperfect complementary pairing allows a single miRNA to have numerous, even hundreds of mRNA targets, thus, globally, potentially regulating the expression of most human proteins [42].

**Figure 1:**
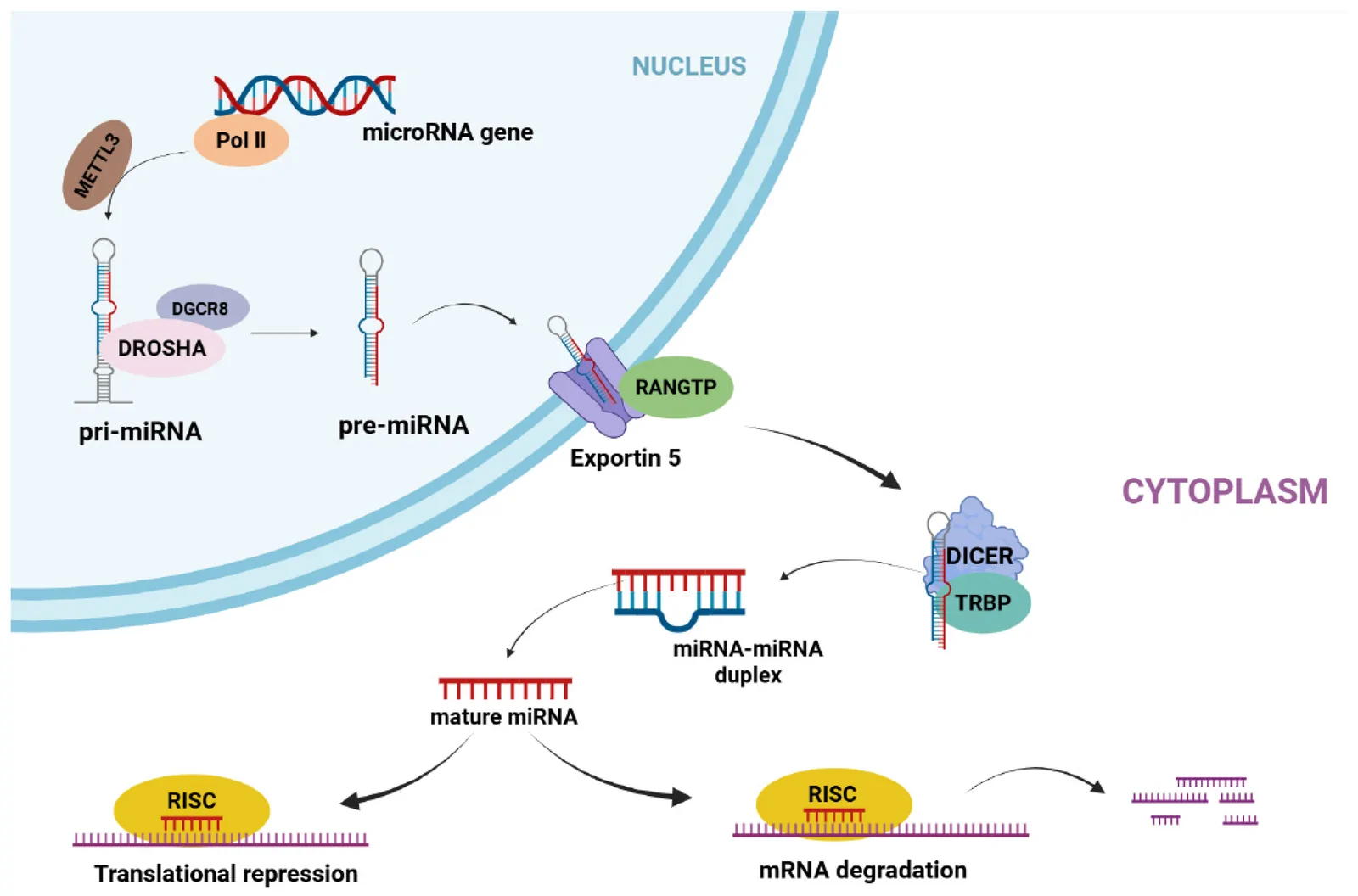
MicroRNA biogenesis diagram illustrating the principal steps of miRNA expression, maturation, and function. Abbreviations in text. Created in BioRender. Mahrous, M. (2026) https://BioRender.com/l41mcpz, access on 17 May 2026.

MiRNA biogenesis comprises a multistep processing pathway [43] (Figure 1). Most miRNAs are transcribed from their genes, although some are encoded within other RNA molecules or introns and further processed to produce their mature form [44,45]. The first transcript produces pri-miRNA, a stem–loop precursor consisting of several thousand nucleotides. The initial processing to form pre-miRNA is completed in the nucleus by a Drosha protein, a ribonuclease, and the DiGeorge Syndrome critical region 8 homolog (DGCR8) complex protein [46,47]. Following pre-miRNA formation, exportin 5 transports pre-miRNA to the cytoplasm, where a Dicer/TRBP complex cleaves the loop structure of pre-miRNA to produce a double-stranded molecule of about 20 bp. One of the miRNA strands (usually the strand with the less stable 5′-end) is subsequently degraded, while the other is preserved to bind with argonaute (AGO) and other proteins to form the RNA-Induced Silencing Complex (RISC). This RISC complex then functions to degrade or suppress mRNA transcripts, thereby silencing certain genes in order to carefully regulate gene expression [45].

Interest in miRNAs has grown in recent literature due to their involvement in paracrine and endocrine secretion, and intercellular signaling [48,49]. After secretion from a cell via the exosomal pathway, miRNAs have been found to transcriptionally regulate gene expression in remote cells. Various pathways have been hypothesized to regulate miRNA sorting into exosomes for secretion, involving sphingomyelinase 2, heterogeneous nuclear ribonucleoprotein A2B1, 3′ post-translational modification of miRNA, and even the RISC itself [50]. Regardless of the mechanism of production, intercellular miRNA communication is key to normal biological function.

### 3.2. Exosomal miRNAs

**Figure 2:**
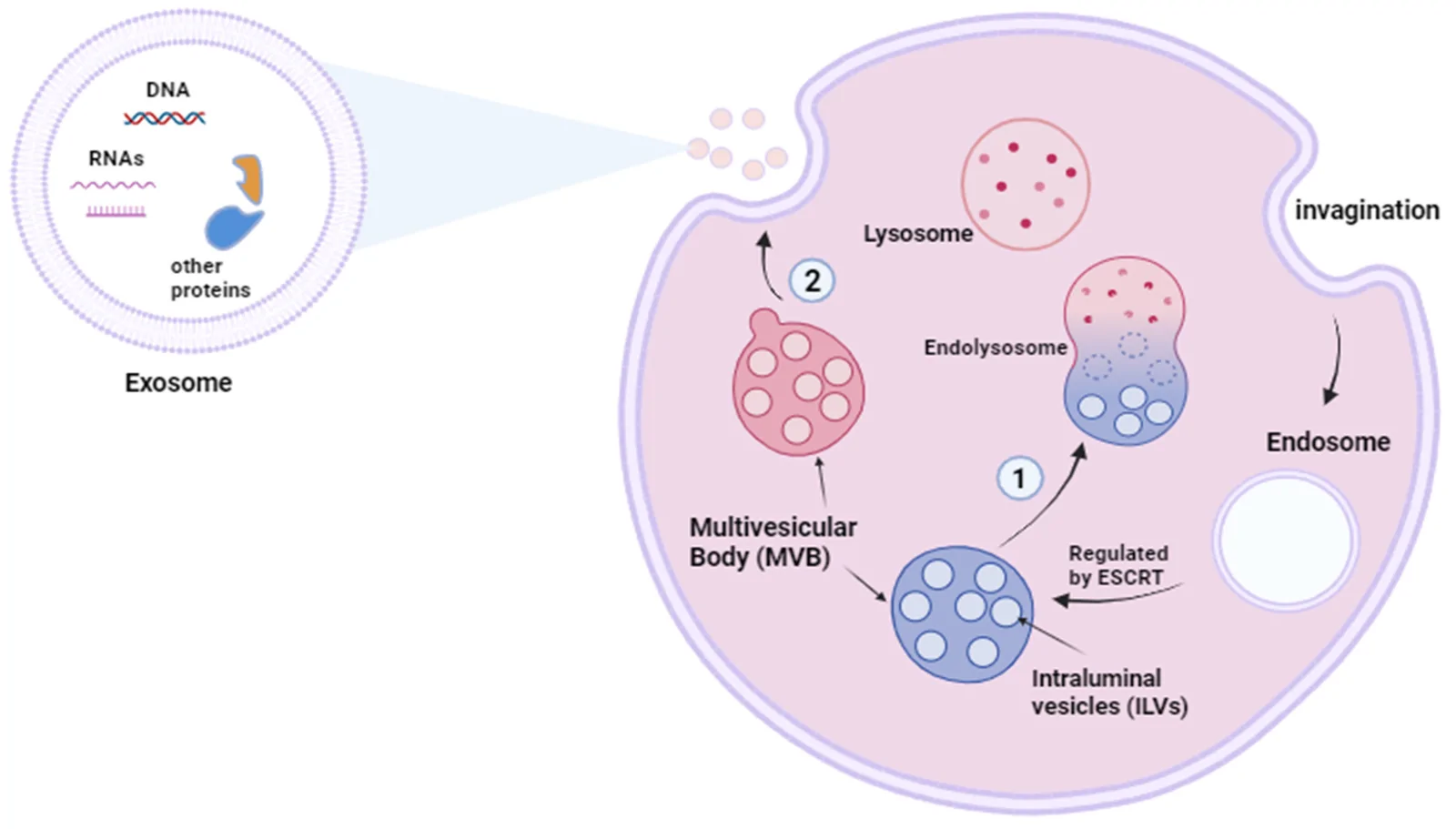
Exosome pathway diagram illustrating exosome formation and release. Abbreviations in text. Created in BioRender. Mahrous, M. (2026) https://BioRender.com/75l8qnm, access on 17 May 2026.

Exosomes are extracellular vesicles generated by the endosomal pathway [51] (Figure 2). Traditionally, a receptor–ligand interaction on the cell surface triggers an invagination of the plasma membrane, resulting in the formation of an early endosome. These vesicles can then combine with an intraluminal vesicle (such as a lysosomal vesicle) to form an intermediate structure called a multivesicular body (MVB) [52]. MVB formation is regulated by a protein multicomplex termed Endosomal Sorting Complex Request for Transport (ESCRT) [53]. MVBs can subsequently undergo two fates. One pathway is to fuse with other MVBs, late endosomes, and/or lysosomes. The other is to migrate to the cell membrane, fuse, and release its contents. Vesicles undergoing this latter pathway are deemed to be exosomes. The exosomal pathway is seen in nearly all cell types [50] (Figure 2).

A unique feature of exosomes is their ability to transport many RNA species, including mRNA, miRNA, circular RNA, and long non-coding RNA. Interestingly, the most numerous cargo molecules in the exosome are, in fact, miRNAs [54,55,56]. Cells utilize the exosomal pathway to transport miRNA molecules to regulate gene expression in nearby cells [50]. Recent research has taken advantage of this property as a method for the therapeutic delivery of drugs [57,58].

### 3.3. MiRNA and the Eye

**Figure 3:**
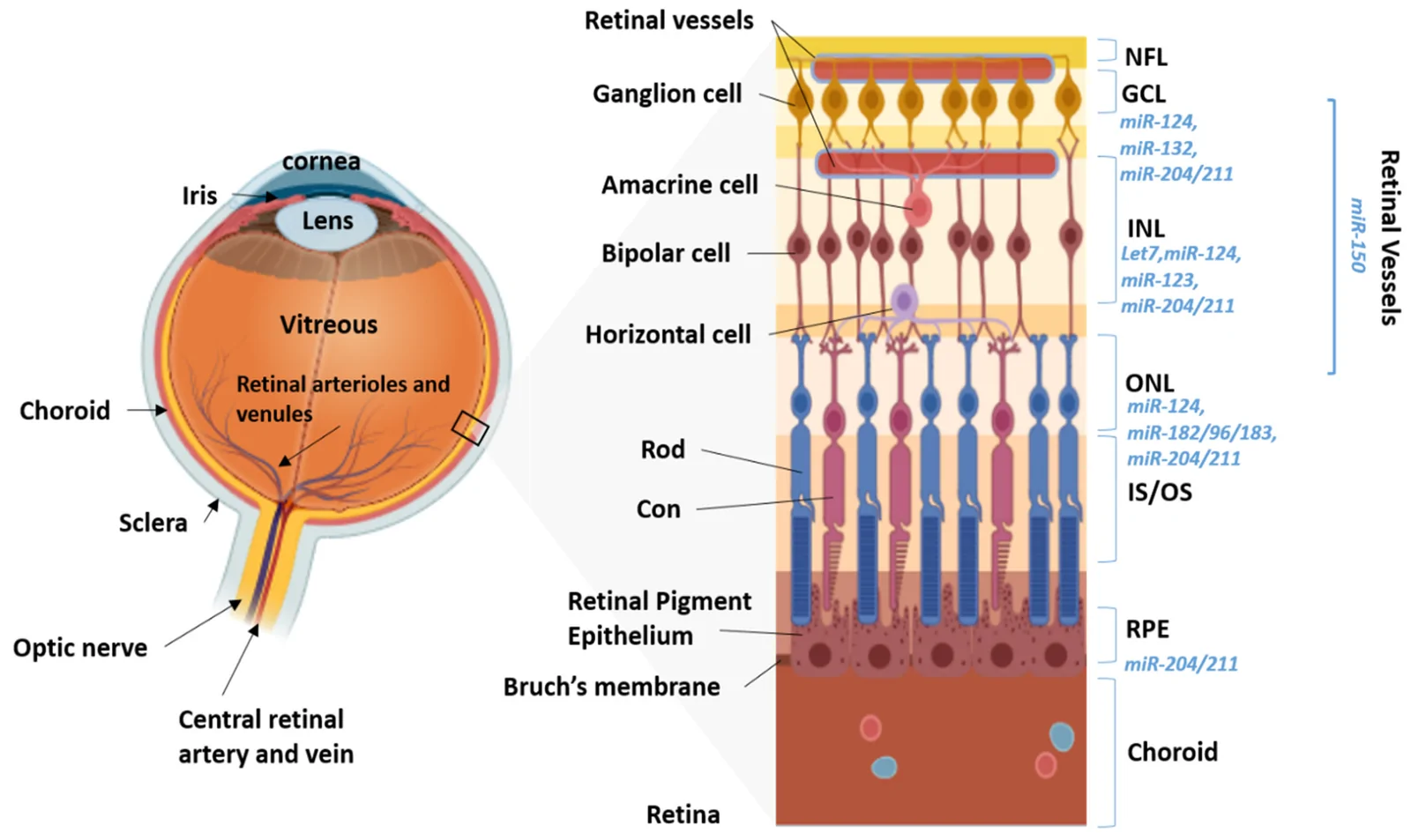
The anatomy of the eye and relevant miRNAs. The schematic diagram illustrates the main structures of the human eye. The schematic representation of the cell types in the neural retina depicts their cellular connections (including ganglion cells, amacrine cells, bipolar cells, and horizontal cells, as well as rod and cone photoreceptors) and supporting cells (Müller cells and RPE), the laminar organization of the nuclear layers (GCL, INL, and ONL), the retinal vasculature, and segments of photoreceptors (IS/OS). The RPE monolayer, with Bruch’s membrane underneath, is located between the neural retina and the choroid complex. MicroRNAs (MiRNAs) that regulate the physiological functions or pathological conditions related to each retinal neuronal and vessel layer, and RPE, are listed next to their histological structures; retinal vessels; GCL, ganglion cell layer; INL, inner nuclear layer; Int, intermediate layer of retinal vessels; IS/OS, inner/outer segments; ONL, outer nuclear layer; RPE, retinal pigment epithelium. Created in BioRender. Mahrous, M. (2026) https://BioRender.com/be6tk51, access on 17 May 2026.

MiRNAs are essential for post-transcriptional gene regulation in many tissues throughout the body, including the eye (Figure 3). Many miRNAs serve a crucial role in the development and maintenance of the retina and are conserved across multiple animal models (Table 1). Among others, miR-124, miR-132, miR-204, and miR-211 have been shown to play important discrete roles in retinal development and homeostasis [59].

MiR-124 is expressed in all retinal neuronal cell layers and is involved in the localization and maintenance of cone photoreceptors through suppression of *Lhx2* transcription [59]. MiR-124 may play a role in the pathogenesis of age-related macular degeneration (AMD) because its levels are found to be depleted from the outer nuclear layer (ONL) and the inner nuclear layer (INL) of the retina in later stages of the disease [60]. Intravitreal administration of miR-124 in mice retinas after photo-oxidative damage not only decreased retinal inflammation and photoreceptor cell death but also improved retinal function [61]. MiR-132, essential for neural synaptic growth in the brain [62], is also expressed in the ganglion cell layer (GCL) and INL of the retina and promotes retinal ganglion cell (RGC) axon formation through downregulation of p250 GTPase-activating protein (p250GAP) [63]. MiR-132 is essential for retinal plasticity, as demonstrated in a lack- and gain-of-function study in mice [64]. Because of its proposed use for ischemic stroke [65], this could also apply to ischemic retinal pathologies. The two most abundant miRNAs in the retinal pigment epithelium (RPE) are miR-204 and miR-211 [66]. They are both found in high amounts in the GCL, ONL, and INL and are critical for directing RPE differentiation and maintaining the RPE epithelial phenotype and function [66]. MiR-204 regulates multiple aspects of eye development, including lens formation, dorso-ventral patterning of the retina, and regulation of eye size and iris shape, as demonstrated in medaka fish [67]. It also regulates differentiation of the RPE, as demonstrated in human fetal RPE (hfRPE) cells [66,67]. Its close relative, miR-211, is crucial for cone cell development, maintenance, and function [68]. Inactivation of miR-211 in mice leads to progressive cone dysfunction and cone loss, without the involvement of rods [68]. The conserved roles of these miRNAs across species demonstrate their crucial role in eye development.

**Table 1:** Essential miRNAs in eye development.

miRNA	Target	Location	Function	Ref.
miR-124a	Lhx2 CCL2	GCL INL ONL	Maturation and survival of dentate gyrus neurons and retinal cone photoreceptors	[69]
miR-132	p250GAP	GCL INL	Promotes axon formation of retinal ganglion cells (RGCs)	[63,64]
miR-204	Meis2, VEGF, VEGFR2 Upregulation CREB5, ELOVL6, TCF12, RAB22A and EMT-associated genes (CDH2, VIM, and SNAI2) Downregulation of RPE-specific genes BEST1, CLDN10, CLDN19, MCT3, RPE65)	GCL INL ONL RPE	Regulates lens formation, dorsoventral patterning of the retina, eye size, RPE differentiation	[66,67,70,71,72,73]
miR-211	Trpm1	GCL INL ONL RPE	Controls metabolism and catabolism of retinal cells	[68]

Lhx2: LIM Homeobox 2; CCL2: C-C motif ligand 2; Meis2: Meis Homeobox 2; Trpm1: transient receptor potential cation channel subfamily M member 1; Spred1: Sprouty Related EVH1 Domain Containing 1; VCAM1: Vascular Cell Adhesion Molecule 1; PIK3R2: Phosphoinositide-3-Kinase Regulatory Subunit 2; GCL: ganglion cell layer; INL: inner nuclear layer; ONL: outer nuclear layer; AMD: Age-related Macular Degeneration; SPRY2: Sprouty homolog 2; RPE: retinal pigment epithelium; VEGF: vascular endothelial-derived growth factor; EC: endothelial cell; c-Kit:CD117 (cluster of differentiation 117) or stem cell growth factor receptor (SCFR); PTEN: phosphatase and tensin homolog; Sema6A: Semaphorin 6A; CNV: choroidal neovascularization.

Dysregulation of specific miRNAs may contribute to abnormalities in proper retinal cell development, maintenance, and function, including retinal blood vessels [74]. Identifying these miRNAs may help uncover the mechanism of angiogenesis of specific retinal vascular pathologies.

Angiogenesis, the formation of new blood vessels from pre-existing ones, is crucial for many tissues’ physiological development and metabolic homeostasis. The mature retina is a highly metabolically active tissue supplied by a complex vascular network of retinal blood vessels and the choriocapillaris [73]. In late embryogenesis, transient retinal hypoxia induces vascular endothelial-derived factor (VEGF) secretion from retinal cells to promote the growth and proliferation of retinal vessels from the optic disc to the retinal periphery [75]. This process has been studied extensively across different animal models [76,77,78,79] and the human fetal retina [80,81,82].

Several mechanisms are responsible for the regulation of retinal vascularization [83], including specific miRNAs. A subset of miRNAs, termed “angiomiRs”, are highly expressed in human vascular endothelial cells and are key regulators of angiogenesis [84]. AngiomiRs affect angiogenesis in many tissues, including the retina, choroid, heart, and lungs. Depending on the angiomiR, it can be categorized as either a pro-angiomiR, which functions by inhibiting negative regulators of angiogenic signaling pathways to promote vessel growth and sprouting, or as an anti-angiomiR, which inhibits positive regulators of the angiogenic process [84]. Some prominent angiomiRs include the miR-17-92 cluster [85], miR-21 [86], miR-23, miR-27 [87], miR-31, miR-126, miR-130a, miR-200 family [88], miR-210, miR-296, miR-378, miR-27b, the let-7 family [89],miR-24 [90], miR-221/222, and miR-320 [59,84].

## 4. MiRNA and Ischemic Retinopathies

Ischemic retinopathies are characterized by abnormal retinal and choroidal vascular growth. These ocular ailments comprise the leading cause of blindness, such as proliferative diabetic retinopathy (PDR), exudative age-related macular degeneration (wAMD), and retinopathy of prematurity (ROP) [91,92,93]. There is increasing evidence of miRNA dysregulation in ischemic retinopathies, and specific miRNAs may serve as biomarkers for disease [94,95].

Among other common pathogenic changes, ischemic retinopathies are all characterized by tissue hypoxia that stimulates retinal VEGF secretion, resulting in vascular hyperpermeability (edema) and abnormal vascular growth [73]. This abnormal neovascular process leads to insufficient oxygen and nutrient supply, as well as waste products [59]. These fragile vessels are also prone to bleeding and predispose to retinal hemorrhages. New vessel growth into the vitreous and inner layers of the retina may also lead to tractional retinal detachment and vision loss [59]. There is increasing evidence of miRNA dysregulation in ischemic retinopathies, and specific miRNAs may serve as biomarkers for disease. In this review, we will focus specifically on those relevant to DR.

Specific angiomiRs are termed “hypoxiamiRs”, as they have been found to disrupt levels of the transcriptional regulator hypoxia-inducible factor (HIF), a VEGF transcriptional regulator that is upregulated in hypoxic conditions. These hypoxia-miRs modulate the HIF/VEGF axis by downregulating HIF1α and VEGF transcription in hypoxic and ischemic conditions [67,78]. Since VEGF stimulates endothelial cell migration, survival, and proliferation, these angiomiRs are crucial for proper vascular growth [78]. Hypoxia and ischemia change the expression profiles of many miRNAs, but only a limited number serve a functional role in angiogenesis [86,87].

Of the angiomiRs mentioned above, those that are also hypoxiamiRs include specific members of the miR-17-92 cluster, miR-31, and miR-126. MiR-17, miR-18a, and miR-20a [67] of the miR-17-92 cluster target HIF1A and VEGF [88,89]; miR-31 targets HIF1A [96]; and miR-126 targets VEGF-A [97]. The miR-17-92 cluster has been studied extensively in the context of lymphoproliferative diseases and is linked to the proliferation, differentiation, and activation of B-cells, T-cells, and monocytes/macrophages mainly through targeting the phosphate and tensin homolog (PTEN) and the transcription factor E2 family [98]. In a mouse model of acute kidney injury, members of this cluster were found to promote angiogenesis after renal ischemia–reperfusion injury through direct repression of thrombospondin 1 (TSP1), a potent antiangiogenic factor [99]. Serum levels of the miR-17-92 cluster have also been found to be elevated in retinoblastoma and proposed as a biomarker for retinoblastoma progression [100].

MiR-31 is a highly conserved miRNA involved in many normal physiological processes such as vascular development, spermatogenesis, embryo implantation, embryonic development, myogenesis, bone homeostasis, autoimmunity, and wound healing [101]. MiR-126 has been studied extensively in autoimmune disorders [102], cancers [103], and atherosclerotic disease [104] due to its cell-specific and strand-specific angiogenic functions [105]. Silencing of the miR-126-3p strand represses but overexpression of the miR-126-5p strand enhances angiogenesis and laser-induced choroidal neovascularization (CNV) in mice [105].

In experimental models of ischemic retinopathies (in vivo and in vitro), these hypoxiamiRs were overexpressed, leading to a decrease in HIF-1α and VEGF levels and a decrease in retinal and choroidal neovascularization. This supports the notion that hypoxic and ischemic conditions alter the delicate balance of tissue-specific and strand-specific miRNAs, potentially contributing to pathologic neovascularization (NV), such as that seen in many ischemic retinopathies [106].

## 5. MiRNAs and Diabetic Retinopathy

To date, several studies have shown that expression and activities of multiple miRNAs are dysregulated in diabetes and DR. These miRNAs are summarized in Table 2 and Table 3 along with the pathways affected. Notably, members of the miR-200 family and the let-7 family may play a role in the pathogenesis of diabetes [107]. The miR-200 family consists of five miRNAs: miR-200a, miR-200b, miR-200c, miR-141, and miR-429, which can be categorized into miR-141/200a and miR-200b/200c/429 based on the homology of their seed sequences [108,109]. In vivo evidence suggests miR-200a induces beta-cell apoptosis by downregulating anti-apoptotic genes caspase inhibitor X-linked inhibitor of apoptosis protein (XIAP) and β-cell chaperone p58IPK, thereby suppressing insulin production [109]. MiR-200 was elevated in diabetic conditions, and its ablation conferred protection against oxidative stress, DNA damage stress, and ER stress-induced beta-cell apoptosis [109]. Let-7 was the first miRNA discovered in humans and is known most notably for its role in tissue differentiation [110,111]. The Let-7 family consists of 12 members in humans (let-7a, let-7b, let-7d, let-7e, let-7c, let-7f, let-7g, let-7i, miR-98, miR-202) and is highly conserved across species in sequence and function. It is involved in a variety of functions throughout the body, including the regulation of insulin production, release, and sensitivity, making it a key player in the pathogenesis of diabetes [112]. Overexpression of let-7 has been shown to lead to features of non-proliferative diabetic retinopathy (NPDR) but also inhibits angiogenesis and choroidal neovascularization in mice, suggesting that let-7 plays a role in early but not late stages of DR [89].

Multiple miRNAs have also been linked to the VEGF pathway and the development of retinal neovascularization in DR (Proliferative Diabetic Retinopathy = PDR). A study on murine models and diabetic patients showed that hyperglycemia and diabetes were associated with the downregulation of plasma levels of miR-200b, which is an inhibitor of VEGF-mediated angiogenesis [113,114]. Downregulated levels in DR lead to the growth of aberrant capillaries with increased vascular permeability, contributing to fluid accumulation in the retinal tissue [113]. Intravitreal injection of an miR-200b mimic prevented VEGF upregulation and VEGF-mediated angiogenesis of leaky blood vessels [113]. Anti-angiogenic miRNAs (miR-106a-5p, miR-20a-3p, miR-20b, and miR-20a-5p) are downregulated in retinal photoreceptor cells under hyperglycemic conditions, a mechanism correlating with aberrant angiogenesis and retinal damage [115].

Furthermore, diabetes has been shown to decrease retinal levels of miR-126, potentially contributing to retinal endothelial injury and diabetic vascular inflammation [32]. A recent study found a positive correlation between circulating levels of miR-15a and severity of type 2 diabetes. In hyperglycemic conditions, there is elevated serum miR-15a due to excessive exosomal secretion by pancreatic beta cells. Retinal Muller cells are the principal glial cells that form the architectural support of the retina. Transport of exosomal miR-15a through the circulation to retinal capillary beds leads to accumulation of miR-15a in retinal cells and promotes apoptotic cell death through the Akt3 pathway [99].

Inflammation also plays an essential role in diabetes, influencing the pathogenesis of various complications, including DR [116,117]. Numerous miRNAs have been identified as key growth factors and cytokine regulators, contributing to retinal inflammation. Notably, miR-146b-3p, miR-152, and miR-200b are associated with this inflammatory process [118,119,120]. MiR-152 is particularly significant as it modulates pro-renin levels, thereby impacting inflammation through the renin–angiotensin system (RAS) [119]. Additionally, miR-152 regulates the expression of VEGF and transforming growth factor-beta (TGF-β) as an effect downstream of the pro-renin receptor (PCR2) complex [119]. The latter, in turn, regulates the levels of miR-200b, which further exacerbates retinal inflammation [121].

Recently, miR-873-5p has been shown to be involved in DR. This miRNA is known to regulate HMOX1, the rate-limiting enzyme in the heme oxygenase reaction pathway [122]. MiR-624-5p and miR-542-3p target prostaglandin-endoperoxide synthase (PTGS2), a rate-limiting enzyme in the arachidonic acid synthesis of prostaglandins [123]. The anti-angiogenic factor Thrombospondin-1 (TSP-1) and its receptor CD36 on endothelial cells are regulated by PTGS2, which may impact DR [124]. Studies revealed that PTGS2 might have a role in ferroptosis-related DR process, which is a new mode of programmed cell death involving lipid peroxide buildup that compromises the integrity of the cell membrane [123]. Pyroptosis, also known as sterile inflammation, is a mechanism of programmed cell death distinguished by reliance on inflammatory caspases and the subsequent release of a multitude of pro-inflammatory factors [125,126,127]. Pyroptosis has been shown to play an important role in DR [125,128]. Studies have identified approximately 20 miRNAs that regulate key pyroptosis-associated genes, including CASP3, TLR4, and GBP2 [129], Given the established role of pyroptosis in DR, these findings warrant further investigation into the potential contribution of these miRNAs to disease pathogenesis.

Recent studies have shown that diabetes-induced vascular senescence is involved in DR induction and progression [130]. MiR-34a is a known regulator of cellular senescence. Studies have demonstrated its key role in promoting vascular senescence in the diabetic retina [131]. This effect was secondary to miR-34a-dependent loss of SIRT1 (silent mating type information regulation 2 homolog) activity and expression [132] and downregulation of mitochondrial endogenous antioxidants, such as thioredoxin reductase 2 (TrxR2) and superoxide dismutase 2 (SOD2), thus directly linking this miRNA to increased retinal endothelial cell susceptibility to oxidative damage and senescence [131,133].

**Table 2:** MiRNA levels in the diabetic retina and in retinal cells exposed to diabetes-relevant stimuli.

miRNA	Intracellular miRNA	Targets	Outcome	Ref.
↓ miR-200b	Diabetic rat retina, human glucose-exposed retinal endothelial cells	VEGF	↑ Angiogenesis ↑ Oxidative Stress	[113,134]
↓ miR-126	Diabetic rat retina, human glucose-exposed retinal endothelial cells	HMGB1, IL-1β, IL-18, and caspase-1, NF-κB/P65	↑ Inflammation	[32]
↑ miR-21	Diabetic rat retina, human retinal microvascular endothelial cells	PPARα	↑ Endothelial cell dysfunction ↑ Inflammation	[135,136]
↑ miR-29b-3p	Human retinal endothelial cells	SIRT1	↑ Retinal cell apoptosis	[137]
↑ miR-34a	Human retinal endothelial cells	SIRT1 TrxR2	↑ Vascular senescence ↑ Oxidative Stress	[131,132,133]
↓ miR-152	Human retinal endothelial cells Rat retina	(pro) renin receptor (PRR)	↑ Diabetic retinopathy	[119]
↑ miR-132	Human retinal pigmented epithelium	Occludin	↑ Vascular permeability	[138]
↓ miR-7a	Endothelial cells of mouse model	IRS-2	↑ Angiogenesis	[139]
↓ miR-15b	Human, HRECs	VEGFA, TNFα, SOCS3	↑ Angiogenesis and inflammation ↑ Insulin resistance	[140,141]
↑ miR-20b-5p	HRMECs, Diabetic rats	BAMBI, THBS1	↑ Angiogenesis	[142,143]
↑ miR-92a-3p	Muller cells of mice	Notch-1	↑ Neuronal death	[144]
↓ miR-133b	HRECs	RhoA, ROCK1	↑ Proliferation of retinal epithelial cells	[145]
↑ miR-383	HRECs	PRDX3	↑ Oxidative stress & apoptosis	[146]
↓ miR-455-5p	Retinal pigment epithelial cells	SOCS3	↑ Oxidative stress, apoptosis and inflammatory response	[147]
↓ miR-451a	Retinal Pigment epithelial cells, Mice retinas	ATF2	↓ Mitochondrial function	[148]
↑ miR-486-3p	Muller cells, Mice	TLR4 p53	↓ Apoptosis and oxidative stress in Muller cells Slows the development of DR	[128,141,142]
↓ miR-26a-5p	Mouse retinal Müller cells, Diabetic mouse	USP14 PTEN	↑ Oxidative stress and inflammation ↑ Neuronal impairment	[149,150]
↑ miR-495 (old)	RGCs of rats	Notch1	↑ Retinal ganglion cell apoptosis	[151]
↑ miR-365	Rats, Retinal neurons	TIMP3, IGF-1	↑ Oxidative stress and apoptosis	[152,153]
↑ miR-423-5p	hRMECs, hRECs	HIPK2	↑ Angiogenesis	[154]
↓ miR-16	HRECs	TNFα, SOCS3	↑ Hyperglycemia induced apoptosis	[141]

↑ increased, ↓ decreased, VEGF, Vascular Endothelial Growth Factor; HMGB1, High-Mobility Group Box 1; IL-1β, Interleukin-1 beta; IL-18, Interleukin-18; NF-κB/p65, Nuclear Factor kappa-light-chain-enhancer of activated B cell p65 subunit; STAT5A, Signal Transducer and Activator of Transcription 5A; p27^Kip1, Cyclin-Dependent Kinase Inhibitor 1B; p57^Kip2, Cyclin-Dependent Kinase Inhibitor 1C; HIF-1α, Hypoxia-Inducible Factor 1-alpha; PPARα, Peroxisome Proliferator-Activated Receptor Alpha; SIRT1, Sirtuin 1; PI3K, Phosphoinositide 3-Kinase; BTG1, B-cell Translocation Gene; JAK/STAT3, Janus Kinase/Signal Transducer and Activator of Transcription 3; NLRP3, NOD-, LRR- and pyrin domain-containing protein 3; NOVA1, Neuro-Oncological Ventral Antigen 1; IRS-2, Insulin Receptor Substrate 2; PI3K, Phosphoinositide 3-Kinase; Akt, serine/threonine kinase 1; TNFα, Tumor Necrosis Factor alpha; SOCS3, Suppressor of Cytokine Signaling 3; BAMBI, BMP and Activin Membrane-Bound Inhibitor; THBS1, Thrombospondin 1; integrins α5/αv, Integrin Subunit Alpha 5/Integrin Subunit Alpha V; Notch-1, Neurogenic locus notch homolog protein 1; RhoA, Ras Homolog Family Member A; ROCK1, Rho-Associated Protein Kinase 1; PRDX3, Peroxiredoxin 3; ATF2, Activating Transcription Factor 2; p53, Tumor Protein P53; TLR4, Toll-Like Receptor 4; NF-κB, Nuclear Factor kappa-light-chain-enhancer of activated B cells; USP14, Ubiquitin-Specific Protease 14; PTEN, Phosphatase and Tensin Homolog; RGCs, Retinal ganglion cells; Notch, Notch Signaling Pathway; TIMP3, Tissue Inhibitor of Metalloproteinases 3; IGF-1, Insulin-Like Growth Factor 1; HIPK2, Homeodomain-Interacting Protein Kinase 2;TNFα, Tumor Necrosis Factor alpha; SOCS3, Suppressor of Cytokine Signaling 3; ADA2, Adenosine Deaminase 2. Downregulated in the fibrovascular membrane in human retinas. Upregulated in Muller cells of mice.

### 5.1. miRNA Polymorphisms and Genetic Susceptibility in DR

miR-related polymorphisms contribute to individual susceptibility to DR by altering miRNA biogenesis, stability, or target recognition. Variants may occur within miRNA genes or miR-binding sites in target genes, affecting pathways involved in inflammation, angiogenesis, oxidative stress, and vascular dysfunction [155]. The miR-146a polymorphism rs2910164 has been associated with susceptibility to sight-threatening DR and may alter NF-κB-mediated inflammatory signaling and VEGF regulation [155]. Similarly, the miR-126 rs4636297 variant has been linked to severe DR, likely through effects on endothelial integrity and angiogenesis [156]. In addition, SNPs within VEGF 3′UTR regions may alter miRNA binding efficiency, influencing VEGF expression and pathological neovascularization [157].

### 5.2. Mechanistic Role of miRNAs in DR Pathogenesis

**Figure 4:**
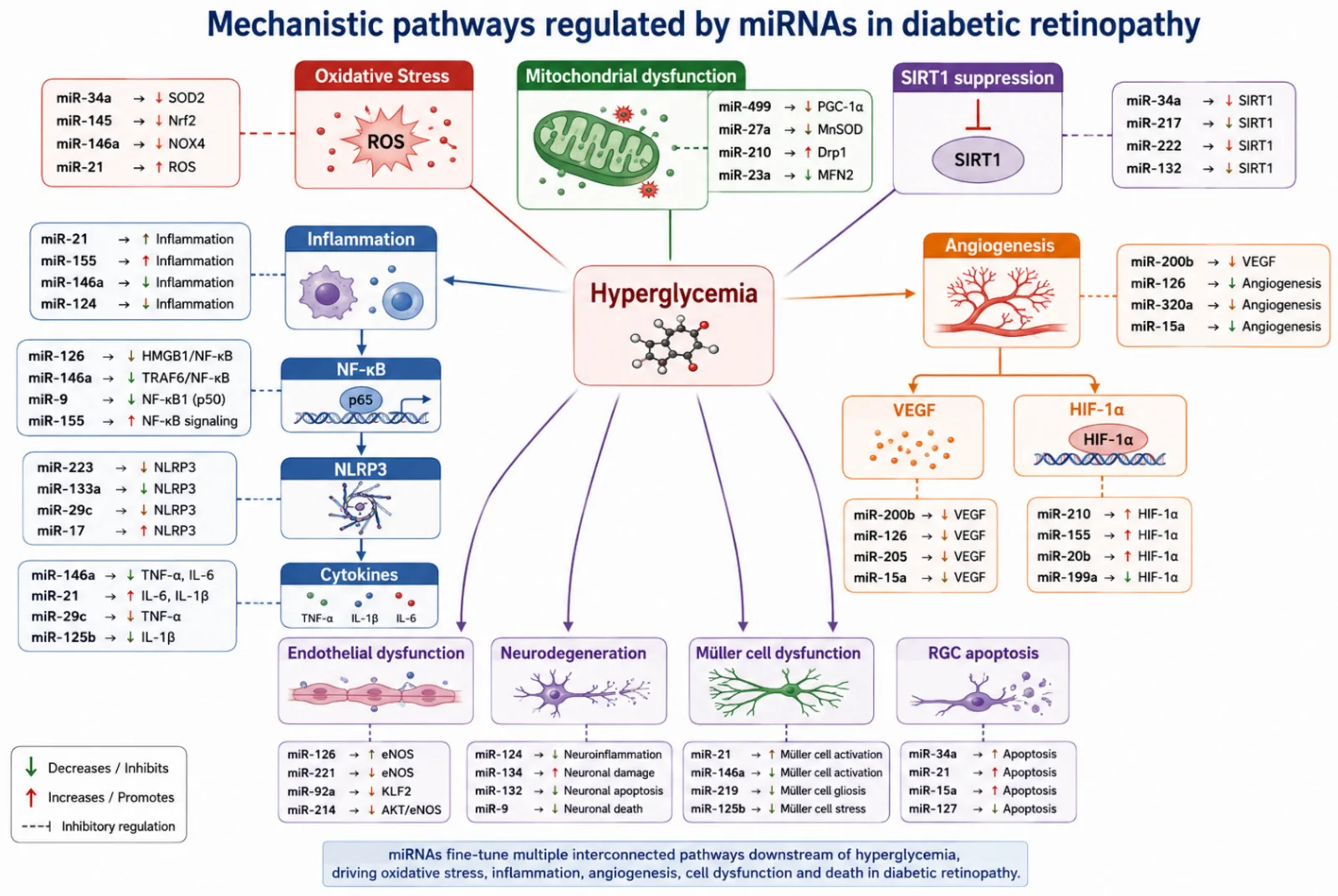
Mechanistic pathways regulated by miRNAs in diabetic retinopathy.

Chronic inflammation is a major contributor to DR, promoting leukostasis, endothelial dysfunction, blood–retinal barrier (BRB) breakdown, and neuronal injury. Hyperglycemia-induced oxidative stress activates inflammatory pathways including NF-κB, NLRP3 inflammasome, HMGB1 signaling, adhesion molecules, and pro-inflammatory cytokines. miRNAs critically regulate these mechanisms [23,158] as summarized in Figure 4.

MiR-146a/b acts as a key anti-inflammatory regulator in DR by suppressing IRAK1 and TRAF6 expression, thereby inhibiting NF-κB signaling and reducing the production of pro-inflammatory cytokines such as IL-1β and TNF-α [159]. It also attenuates ICAM-1 expression, decreases leukostasis, and suppresses inflammasome activation [160]. Reduced miR-146a/b expression contributes to persistent retinal inflammation, vascular permeability, and progressive retinal injury in DR.

MiR-21 primarily functions as a pro-inflammatory miRNA in DR. It enhances NF-κB-associated inflammatory signaling, promotes microglial activation, and increases TNF-α-mediated inflammatory responses, thereby contributing to retinal inflammation, vascular dysfunction, and neuronal injury [121].

miR-126 exerts vasculoprotective, anti-inflammatory, and anti-angiogenic effects in DR by maintaining endothelial homeostasis and preserving blood–retinal barrier (BRB) integrity. It suppresses endothelial activation, leukostasis, vascular permeability, and inflammatory signaling while regulating VEGF/HIF-1α and PI3K/Akt pathways. Reduced miR-126 expression contributes to BRB breakdown, vascular leakage, inflammation, and endothelial dysfunction [161].

miR-30a attenuates retinal inflammation and cellular injury in DR by suppressing NLRP3 inflammasome activation and reducing oxidative stress- and apoptosis-associated signaling pathways. Through these effects, miR-30a limits IL-1β and IL-18 production and protects against cytokine-mediated retinal damage [162].

miR-152 appears to function as an anti-inflammatory regulator in DR, as decreased miR-152 expression is associated with enhanced NLRP3 inflammasome activation and increased retinal inflammation [163].

miR-34a is a major pro-oxidative and pro-apoptotic miRNA in DR. It promotes ROS accumulation, mitochondrial dysfunction, apoptosis, and ferroptosis by suppressing antioxidant and survival pathways, particularly through inhibition of the SIRT1/FOXO signaling axis. miR-34a also indirectly reduces SOD2-mediated antioxidant defense, thereby exacerbating oxidative stress, mitochondrial fragmentation, and retinal cell injury [133].

miR-383 contributes to oxidative stress and inflammatory injury in DR by enhancing ROS accumulation and ferroptotic signaling pathways. Dysregulated miR-383 is associated with increased oxidative damage and retinal inflammation [148].

miR-451a functions as an antioxidant and cytoprotective miRNA in DR by preserving mitochondrial homeostasis, reducing oxidative stress, and enhancing antioxidant defense mechanisms. It may improve SOD2 and TrxR2 activity and exerts anti-ferroptotic effects that protect retinal cells from oxidative injury [150].

miR-26a-5p exerts antioxidant and anti-ferroptotic effects in DR by enhancing SIRT1-associated antioxidant signaling and strengthening cellular resistance to oxidative stress. It supports mitochondrial protection and reduces oxidative retinal injury [164].

miR-200b acts as a potent anti-angiogenic miRNA in DR by directly targeting VEGF mRNA and suppressing VEGF/HIF-1α-mediated pathological angiogenesis and vascular hyperpermeability. It also modulates Notch-associated angiogenic signaling and helps preserve endothelial barrier function [165].

miR-92a negatively regulates endothelial repair and angiogenic homeostasis in DR. Increased miR-92a expression impairs PI3K/Akt-associated endothelial repair mechanisms, exacerbates vascular dysfunction, and promotes BRB breakdown and retinal vascular leakage [166].

### 5.3. MiRNAs as Biomarkers in DR

MiRNAs can be classified as intracellular or extracellular. Multiple studies have been conducted that measured miRNA levels in retinal cells, endothelial cells, aqueous humor, vitreous humor, and serum of diabetic animal models as well as diabetic patients [94,167,168,169]. These studies support the idea that miRNAs act as hormone-like molecules that travel through the bloodstream or other biological fluids via exosomes to promote maladaptive responses to hyperglycemia; therefore, they have the potential to serve as novel biomarkers of disease progression. Additionally, their role in paracrine signaling makes them a potential candidate to serve as treatment options via delivery by exosomes and/or intraocular injection.

Intracellular miRNAs that are dysregulated in DR are summarized in Table 2. Studies examining miRNA levels in retinal and endothelial cells were mostly done on animal models due to the difficulty of performing retina biopsy on diabetic patients. MiRNAs that were found to be downregulated in animal models with DR included miR-106a-5p, miR-20a-3p, miR-20a-5p, miR-20b [170], miR-200b [171], miR-126 [172,173], miR-222 [174], miR-7a [139], and miR-139-5p [175]. Most of these miRNAs target the VEGF pathways and have been shown to play a role in regulating angiogenesis. Therefore, their downregulation could contribute to abnormal neovascularization, as seen in PDR [121]. MiRNAs that were upregulated in DR included miR-21 [176], miR-29b-3p [137], miR-34a [132,177] and miR-183 [178,179]. Upregulation of miR-21 and miR-29b-3p was associated with endothelial cell dysfunction and retinal cell apoptosis [94,137], while upregulation of miR-183 was associated with increased angiogenesis [178].

Due to the present difficulty in assessing intracellular miRNAs in clinical practice, it is important to identify extracellular miRNAs that may serve as biomarkers for DR, as these are more easily collected and analyzed. Multiple studies have been conducted on patients with DR to identify dysregulated miRNAs (summarized in Table 3). Most of the identified miRNAs play a role in pathways that regulate angiogenesis and endothelial cell dysfunction. Some notable miRNAs include miR-221, which multiple studies have shown to be upregulated in the plasma of patients with DR [95,180,181,182,183]. MiR-210 was also elevated in the plasma of patients with DR, while miR-126, miR-146a, and miR-200b were downregulated in serum [113,114,184,185]. In terms of intraocular miRNAs, miR-16, miR-92, miR-146a, and miR-146b-3p were found to be downregulated [94,120,141,186,187], while miR-21, miR-93, and miR-423-5p were upregulated in vitreous humor of DR patients [95,135,188,189,190]. Studies have shown that miR-16 provided over 90% sensitivity and specificity in distinguishing patients with DR from patients without DR [141,186]. Lastly, certain miRNAs such as let-7b, let-7a-5p, miR-320b, miR-762, and miR-4488 are dysregulated in all 3 fluid compartments [186]. MiR-let-7b was shown to be upregulated in aqueous and vitreous while being downregulated in plasma; miR-320b was upregulated in all three fluid compartments; and miR-762 and miR-4488 were either upregulated or downregulated in aqueous humor, vitreous humor, and plasma depending on the severity of DR [186]. Moreover, there is significant and progressive downregulation of circulating and intraocular miR-150-5p and miR-329 in patients with DR [186,191,192]. Interpretation of circulating and intraocular miRNA profiles is further complicated by methodological heterogeneity among studies. Differences in sample source (serum, plasma, vitreous, aqueous humor), RNA isolation protocols, sequencing platforms, normalization methods, patient demographics, diabetes duration, and DR stage may significantly influence reported miRNA expression patterns. Standardization of experimental and analytical approaches will therefore be essential before miRNAs can be reliably translated into clinical biomarkers.

**Table 3:** Extracellular miRNA in Diabetic Retinopathy.

miRNA	Location	Targets	Outcome	Ref.
↓ miR-146b-3p	Human eyes	ADA2	↑ Inflammation	[120]
↓ miR-92a	Human retinas	integrins α5/αv	↑ Fibrovascular membrane formation	[193]
↓ miR-106a-5p, miR-20a-3p, miR-20a-5p, miR-20b	Mice retina	VEGF	↑ Angiogenesis	[115]
↓ miR-222	Rabbit retina	STAT5A, p27Kip1, p57Kip2	↑ Retinal cell damage	[174]
↑ miR-30a	Diabetic rats	NLRP3	↑ Neuroinflammation	[168]
↓ miR-203a-3p	Rat retina	VEGF and HIF-1a	↓ Angiogenesis	[194]
↑ miR-183	Rat retina	PI3K/Akt/VEGF BTG1	↑ Angiogenesis	[178]
↓ miR-138-5p	Diabetic rats	NOVA1	↑ Proliferation of retinal endothelial cells and pericytes	[195]
↑ miR-210	Serum	PTP1B, GPD2	Regulates angiogenesis	[196,197]
↑ miR-221	Serum	TIMP3	Induces vessel leakage and endothelial cell dysfunction	[187,198]
↓ miR-126	Plasma, Vitreous Humor	PLK4,VEGF, Ang-1, VCAM-1, IRS-1	Suppresses angiogenesis	[172,173,199,200]
↓ miR-200b	Serum	VEGFA	Induces Angiogenesis	[113,114]
↓ miR-92	Vitreous	CD34+ progenitor differentiation genes, Per2 gene	Maintains CD34+ cells	[94]
miR-93 *	serum	SIRT1, VEGF	Decreases retinal vascular permeability Mitigate inflammation and oxidative stress	[190,201]
↑miR-21	Plasma	PPARa	Induces Inflammation	[136,202]
↑ miR-320, miR-320a, miR-320b	Aqueous humor, vitreous	neuropilin1, VEGF; insulin–PI3K signaling pathways, thrombospondin-1	Regulates angiogenesis	[94,203,204]
miR-27a **	Serum, vitreous	TLR4, IRAK4	Modulates the inflammatory response	[203,205]
↑ Let-7a-5p, Let-7p; ↓ Let-7c (vitreous)	Serum, Aqueous humor, vitreous	Glucose metabolism	Regulates angiogenesis	[89,94,181]
↓ miR-150-5p	plasma	ELK1	Exacerbates T2D induced-photoreceptor apoptosis	[206,207]
↓ miR-17-3p	Serum	STAT1,	Its upregulation ameliorates inflammation and oxidative stress	[208,209]
↓ miR-26a-5p	serum	USP14/NF-κB, PTEN	Its downregulation promotes HG-triggered Müller cell dysfunction, oxidative stress and inflammation.	[149,150,210]
↓ miR-495	Plasma	Notch/PTEN/Akt pathway	Its downregulation alleviates retinal cell apoptosis and neurodegeneration.	[151,211]
↓ miR-15a, ↓ miR-15b	Plasma	ASM, VEGF	Its downregulation promotes angiogenesis and inflammation	[140,212,213]
↓ miR-329	Plasma	CD146	Its downregulation promotes angiogenesis	[191]

↑ increased, ↓ decreased, PTP1B, Protein Tyrosine Phosphatase 1B; GPD2, Glycerol-3-Phosphate Dehydrogenase 2; TIMP3, Tissue Inhibitor of Metalloproteinases 3; PLK4, Polo-Like Kinase 4; VEGF, Vascular Endothelial Growth Factor; Ang-1, Angiopoietin-1; VCAM-1, Vascular Cell Adhesion Molecule 1; IRS-1, Insulin Receptor Substrate 1; SIRT1, Sirtuin 1; PPARa, Peroxisome Proliferator-Activated Receptor Alpha; PI3K, Phosphoinositide 3-Kinase; T1DM, Type 1 Diabetes Mellitus; TLR4, Toll-Like Receptor 4; IRAK4, Interleukin-1 Receptor-Associated Kinase 4; ELK1, ETS-Like Protein 1. STAT1, Signal Transducer and Activator of Transcription 1; USP14, Ubiquitin-Specific Protease 14; NF-κB, Nuclear Factor kappa-light-chain-enhancer of activated B cells; PTEN, Phosphatase and Tensin Homolog; Notch, Notch Signaling Pathway; Akt, serine/threonine kinase 1; ASM, Acid Sphingomyelinase. * Downregulated in patients’ serum, upregulated in rats’ retinas. ** Has variable levels.

### 5.4. MiRNAs with Beneficial Effects on DR

Some exosomal miRNAs secreted by mesenchymal stem cells (MSCs) have been shown to attenuate the aberrant changes in DR. Current studies show that delivery of miR-216a, miR-200b, miR-126, and miR-222 can reduce angiogenesis and inflammation, and promote retinal repair in animal models [32,113,174,214] (Table 4). An experiment by Zhang et al. demonstrated that miR-126 delivery via MCS-derived exosomes effectively downregulated the expression of the inflammatory cytokine HMGB1, dampened the inflammatory signaling pathways that led to NLRP3 inflammasome activation, and reduced NF-κB/P65 protein levels in diabetic rats and human retinal endothelial cells (HRECs) cultured in high-glucose media [32]. This supports that miR-126 may be essential in maintaining retinal endothelial cell vascular integrity and ameliorating the inflammatory response to hyperglycemia [32]. Another recent study found that retinal miR-222 is significantly decreased in streptozotocin-induced diabetic rabbits, and low levels were associated with the development of severe retinal damage consistent with extensive hemorrhages, retinal detachment, and macular edema [174]. Administration of miR-222 via exosomes from adipose-derived mesenchymal stem cells (MSC) via intraocular and subcutaneous injection facilitated retinal tissue repair and restored the cellular components of the retina to normal over a 12-week period [174]. These studies provide promising support for the use of miRNAs in the treatment of DR.

## 6. Potential Clinical Applications

To date, DR remains the leading cause of blindness among adults, thus urging the need to identify new diagnostic and therapeutic tools. There is a significant amount of work supporting the role of miRNAs in DR pathogenesis; however, their relative impact on DR diagnosis or treatment is still under investigation. Using miRNAs as a biomarker for DR may improve its screening and diagnosis in areas with a shortage of eye care specialists or regions that lack complex imaging modalities needed to visualize the retina. However, current studies involve small sample sizes, and future research involving larger studies needs to be conducted to identify possible markers [217].

Current treatments for DR include laser photocoagulation therapy to ablate aberrant blood vessels, intravitreal anti-VEGF treatment and intraocular corticosteroid implants to treat macular edema, and intraocular surgery to remove scar tissue that is obscuring vision. Limitations of current treatment options include unsatisfactory efficacy and high treatment burden [218]. The intravitreal anti-VEGF injection is commonly used among the various non-surgical treatments for DR; however, about 15–25% of patients with DR do not respond well to anti-VEGF therapy [219,220,221]. Therefore, there is a growing need for alternative treatment options that provide a more targeted approach.

MiRNAs present an intriguing therapeutic intervention option because they can control the expression of several genes, are simple to produce, and have low toxicity [211]. MiRNA manipulation generally follows two principal strategies, depending on whether the goal is to suppress an overexpressed miRNA (loss of function) or to restore the function of a downregulated one (gain of function).

Loss-of-function modulation of miRNAs is primarily accomplished through:

(i) Antagomirs (anti-miRs) that are chemically synthesized single-stranded oligonucleotides designed to bind and inactivate specific miRNAs [222]. They have been successfully utilized in preclinical models of DR to silence upregulated miRNAs that drive inflammation and vascular problems. For example, inhibiting miR-21, which is associated with proinflammatory and profibrotic signals, has been shown to reduce NF-κB activation and retinal vascular leakage in streptozotocin (STZ)-induced diabetic rats [223]. (ii) miRNA sponges are synthetic transcripts containing multiple tandem binding sites that are complementary to a particular miRNA or miRNA family, functioning as competitive inhibitors [224]. These constructs have been delivered via adeno-associated virus (AAV) vectors in ocular disease models [225,226]. In experimental DR, sponge-based inhibition of miR-15a/16, which is involved in endothelial apoptosis and angiogenesis, led to improved retinal vascular integrity [227]. (iii) A miRNA mask is a specially designed single-stranded molecule that blocks miRNA from binding to its target messenger RNA (mRNA). This effect results by directly binding of the mask to the target mRNA at the miRNA attachment site, effectively “masking” that region [228]. This prevents the miRNA from suppressing protein production, allowing the mRNA to be translated more efficiently and increasing the expression of the target gene. (iv) Small-molecule inhibitors, identified through bioinformatics or experimental screening of active compounds, work by disrupting proteins involved in miRNA biogenesis or by preventing miR–target interactions through binding to specific secondary structures of the miRNA [229].

Restoration of function strategies mainly depend on using either chemically synthesized double-stranded miRNA mimics or viral vectors to enhance miRNA expression. These synthetic mimics are not identical to endogenous miRNA duplexes; the guide (sense) strand is modified to be fully complementary to the mature miRNA for increased stability [229]. Additionally, chemical modifications are made to inactivate the sense strand, as it no longer reflects the natural sequence and could otherwise cause unintended effects. miRNA mimics can be designed to silence a specific mRNA target or include multiple miRNA sequences, allowing the simultaneous regulation of different genes [230]. In contrast, viral vector-based miRNA overexpression involves using integrating vectors that carry short hairpin RNAs (shRNAs) under the control of RNA polymerase III promoters [231]. Once delivered, these vectors enable stable and long-term expression of the desired miRNA. Mesenchymal stem cells are multipotent cells that have the potential to transform into many cell types and can be isolated from a variety of tissues such as umbilical cord, bone marrow, and adipose tissue, making them a feasible source for experimentation and possible clinical application [198]. Exosomes are currently recognized as an important shuttle for cellular communication, as they can encapsulate and deliver various genetic materials (mRNAs, miRNAs, and proteins) to receptor cells to regulate various cellular processes. The use of MSC-derived exosomes enriched with beneficial miRNAs such as MiR-126 and MiR-222 may be a new strategy for the treatment of DR [26,121].

Several miRNAs such as miR-16, miR-216a, miR-200b, miR-126, miR-222, and miR-329 have been shown to reduce angiogenesis, control inflammation and promote retinal cell repair, making them potential therapies. The use of miR-200 mimics to downregulate VEGF release in ischemic retinopathies may be a potential treatment option for managing DR and macular degeneration, and retinopathy of prematurity. MiR-329 has also been reported to have anti-angiogenic characteristics by inhibiting the production of CD146, an adhesion protein that functions as a VEGF co-receptor. As a result, miR-329-based therapy demonstrated dramatically reduced retinal neovascularization in animal models of pathological angiogenesis [215]. Currently, treatments for DR focus on mitigating neovascularization. The ability of miR-222 to reverse retinal damage provides the potential to develop a revolutionary treatment that would not only control but also reverse retinal damage. Subcutaneous and intraocular administration of MSC exosomes may represent a better route of delivery, as these routes were associated with improved retinal repair in STZ-induced diabetic rabbits [174]. While these are promising studies, they were performed mostly in animal models. Therefore, more work needs to be conducted using human-derived MSC exosomes to cargo-specific miRNAs in human ocular tissues.

## 7. Limitations of the Therapeutic Use of miRNAs

Although pre-clinical studies have shown promising outcomes, miR-based therapies have struggled to move into clinical use due to several ongoing challenges. Only a few of the many miR-targeting compounds have progressed to clinical trials. Key hurdles include identifying the right molecular targets, ensuring that miRNA inhibitors remain stable in biological fluids, and improving their precision to avoid off-target effects and unintended changes in gene expression [232]. For instance, MRX34, a synthetic miR-34a mimic, was used in a clinical trial, and due to its broad activity and lack of tumor specificity, led to serious off-target effects, highlighting the need for better delivery systems and tighter control of miRNA mimic activity in future therapies [233]. The inherent pleiotropy of miRNAs also raises concerns about off-target effects, underscoring the importance of rigorous safety evaluations, particularly for long-term use. Moreover, effective delivery across ocular barriers such as the inner blood–retinal barrier remains a critical hurdle. To address these issues, advanced delivery strategies, including nanoparticle-based carriers [234], exosomes, and tissue-specific viral vectors [235], are currently being explored to enhance retinal targeting while limiting systemic exposure. Nanotechnology has significantly advanced drug delivery by offering new methods to boost effectiveness and reduce side effects. Central to these innovations are nanoparticles, tiny structures between 1 and 100 nanometers in size that can carry therapeutic agents like miRNAs directly to the retina and avoid contact with other eye tissues [236]. Their unique properties allow drugs to be encapsulated and delivered with greater precision. Exosomes, often lipid-based, offer a promising platform for drug and gene delivery. They can be loaded with miRNAs, are readily absorbed by cells, do not trigger immune responses, and can cross critical barriers such as the blood–brain and blood–retinal barriers [94,237]. As, nanoscale delivery systems, engineered exosomes are gaining significant attention for targeted miRNA therapy. Given their broad regulatory roles across physiological and pathological pathways, miRNA-based therapies continue to hold substantial therapeutic potential, provided these delivery and safety challenges can be effectively addressed [238]. Despite substantial preclinical evidence supporting the therapeutic potential of miRNAs modulation, to date no miRNA-based therapy has yet demonstrated clinical efficacy in late-stage trials or received regulatory approval for DR. Consequently, miRNA-based interventions remain experimental, and significant challenges must be overcome before clinical translation can be achieved.

An additional challenge for the clinical translation of miRNA-based biomarkers and therapeutics is the tissue- and disease stage-specific nature of miRNA expression. The expression and functional effects of individual miRNAs may differ among retinal cell types, circulating biofluids, and the various stages of DR, due to the dynamic progression of the disease. Consequently, careful consideration of tissue specificity and disease stage will be essential for the identification of reliable biomarkers and the development of personalized miRNA-based therapeutic strategies.

## 8. Conclusions

Dysregulated miRNAs expression and function are closely associated with diabetes pathogenesis and the induction and progression of its complications, including DR. Therefore, miRNAs can be a novel and potentially valuable biomarker for early recognition and treatment of many diseases, including DR. They participate in various gene regulatory pathways to modulate disease progression. The stability of these molecules in biological fluids such as serum, plasma, vitreous, tears, and aqueous humor allows for easy collection and analysis in the various clinical specimens. Several miRNAs are dysregulated in patients with DR, making these molecules potential disease biomarkers. Additionally, miRNAs have been shown to regulate target genes responsible for inflammation and angiogenic factors involved in the pathogenesis of DR and its complications (diabetic macular edema and proliferative diabetic retinopathy).

Interpretation of miRNA dysregulation in DR remains challenging due to substantial variability among studies. Differences in species, duration of diabetes, glycemic severity, retinal cell types analyzed, sequencing platforms, normalization strategies, and sample processing may all contribute to inconsistent findings across reports. Additionally, some miRNAs exhibit tissue-specific or stage-specific expression patterns, complicating direct comparisons between studies.

Future treatment strategies can focus on delivering beneficial miRNAs exogenously through exosomes, allowing a more targeted approach for retinopathy treatment compared to current methods. Although much more research needs to be conducted on the use of miRNAs as biomarkers and the development of exosome-based drug delivery systems, exosomal miRNAs hold promising potential to aid in the prevention, diagnosis, and treatment of DR.

## Figures and Tables

**Table 4:** Beneficial miRNA in Diabetic Retinopathy.

Table 4: Beneficial miRNAs Delivered	Delivery Method	Tissue	Pathway Affected	Ref.
miR-16	Transfection	HREC	↓ TNFα and SOCS3 to prevent hyperglycemia-induced apoptosis	[141]
miR-216a	Transfection	HRMEC	↓ NOS2/JAK/STAT axis to reduce inflammation	[214]
miR-200b	Transfection, IV injection	Diabetic mouse retina, RMECs	↓ VEGF and glucose-induced vessel permeability	[113]
miR-126	Human umbilical cord-derived MSC exosomes	Diabetic rats, HREC	↓ HMGB1 to reduce inflammation	[32]
miR-222	Mesenchymal stem cell exosomes	Rabbit Retina	↑ Retinal repair	[174]
miR-329	Transfection	HUVEC	↓ VEGF through inhibition of CD146	[215]
microRNA-17-3p	Injection of hucMSC-derived exosomes	Diabetic mice	↑ STAT1 and ↓ VEGF	[209]
miR-146a	intravitreal injection of Lentiviruses expressing miR-146a	Diabetic rats	↓ NF-κB, ICAM1	[216]

↑ increased, ↓ decreased HREC: Human Retinal Endothelial Cell; TNFα, Tumor Necrosis Factor alpha; SOCS3, suppressor of cytokine signaling 3; HRMEC, Human retinal microvascular endothelial cell; NOS2, Nitric Oxide Synthase 2; JAK, Janus Kinase; STAT, Signal Transducer and Activator of Transcription; RMEC, Retinal Microvascular Endothelial Cell; HUVEC, Human Umbilical Vein Endothelial Cell; VEGF, Vascular Endothelial Growth Factor; HMGB1, High-Mobility Group Box 1; hucMSCs, Human Umbilical Cord Mesenchymal Stem Cells; NF-κB, Nuclear Factor kappa-light-chain-enhancer of activated B cells; ICAM1, Intercellular Adhesion Molecule 1.

## Data Availability

No new data were created or analyzed in this study.
